# A bibliometric analysis on the progress of myocardial bridge from 1980 to 2022

**DOI:** 10.3389/fcvm.2022.1051383

**Published:** 2023-01-06

**Authors:** Liang Chen, Wen-Yuan Yu, Rui Liu, Ming-Xin Gao, Bo-Lin Wang, Xiao-Hang Ding, Yang Yu

**Affiliations:** Department of Cardiac Surgery, Beijing Anzhen Hospital, Capital Medical University, Beijing, China

**Keywords:** myocardial bridge, review, bibliometric, atherosclerosis, management

## Abstract

**Introduction:**

Although the vast majority of patients with a myocardial bridge (MB) are asymptomatic, the anomaly was found to be associated with stable or unstable angina, vasospastic angina, acute coronary syndrome, and even malignant arrhythmias and sudden cardiac death in some cases.

**Methods:**

By retrieving the relevant literature on MB from 1 January 1980 to 31 July 2022 from the Web of Science Core Collection (WoSCC) database, we used the bibliometric tools, including CiteSpace, VOS viewer, and alluvial generator, to visualize the scientific achievements on MB.

**Results:**

A total of 630 articles were included. The number of published articles was in a fluctuating growth trend. These publications came from 37 contries, led by the USA and China. The leading country on MB was the United States, the leading position among institutions was Stanford University, and the most productive researcher on MB was Jennifer A. Tremmel. After analysis, the most common keywords were myocardial bridge, mortality, coronary angiography, descending coronary artery, and sudden death.

**Conclusion:**

Our findings can aid researchers in understanding the current state of MB research and in choosing fresh lines of inquiry for forthcoming investigations. Prevalence and prognosis, mechanism atherosclerosis, hemodynamic significance, and molecular autops will likely become the focus of future research. In addition, more studies and cooperations are still needed worldwide.

## Introduction

Anatomically speaking, a myocardial bridge (MB) is a congenital anomaly that includes systolic arterial compression and a length of tunneled artery beneath a portion of the myocardium. Although case studies suggest that MB may clinically show as angina, acute coronary syndrome, or malignant arrhythmias potentially leading to sudden cardiac death, the majority of MBs are asymptomatic (1–3). Unlike an atherosclerotic cardiovascular disease, the clinical manifestation of MB often occurs in young patients (4). However, since there are presently no guidelines for the best therapeutic approach, centers and specialists have different perspectives on how to care for patients with MB.

To our knowledge, there are no bibliometric analyses of MB currently. Our goal was to use this approach to assess the state of the art and new directions in MB-related study and to offer an in-depth analysis of the field's state of development for researchers to refer to for future work.

## Methods and materials

Web of Science Core Collection (WoSCC) was used to collect research on MB in the study. The search parameters were as follows: TS = (“myocardial bridge^*^”); time span: 1 January 1980 to 31 July 2022; with language type: English, literature type: article; and index: sci-expanded, SSCI. As shown in Supplementary Table 1, we discovered that the number of publications each year related to MB was quite a few before 1980. Because of this, we decided to start the retrieval year in 1980.

Then, using 630 articles, we created a clustered network. By performing co-citation analysis and burst identification during the last 43 years, three types of bibliometric analysis tools VOS viewer 1.6.18, CiteSpace 6.1.R3 Advanced and alluvial generator (http://www.mapequation.org/apps/AlluvialGenerator.html) were utilized to mine the intellectual foundation and boundaries of MB research. To guarantee the correctness and dependability of the data, Liang C and Wen-Yuan Y did data extraction and analysis management, respectively.

CiteSpace was used to discover the co-authorship network of authors, countries, and institutions. Each point in the graphs represented one element, such as an author, a country, or an institution, whose size was indicated by the size of the point. In addition, the interconnections between the points reflected the relationship of co-citation, and the thickness of the cooperation appeared to increase with the number of interconnections, representing the strength of the link. We set CiteSpace's parameters as Time Slicing (1980–2022), with 7 years per slice and top 50% criteria.

The VOS viewer was used to display the co-citation analysis of references, journals, and authors, as well as the co-occurrence of keywords. Different points in the co-citation maps stand in for various components (co-cited references, journals, and authors), and the size of the points is proportional to the number of citations the articles have received (5). Co-citation connections are shown by the lines connecting the spots (6, 7). Various clusters or years are represented by different colored points and lines(8). In order to reflect the same study subject or direction, we also utilized CiteSpace to create a network map of co-citation clusters and a timeline view of co-citation clusters.

To comprehend the structural changes in co-cited references and investigate the consistently significant research throughout the previous 6 years, we employed an alluvial diagram. Our study's alluvial flow map was constructed using information obtained from CiteSpace. An alluvial generator was used to directly import the networks of co-cited references that were first created in CiteSpace by g-index with a scale factor of 25 in the most recent 6 years (2017–2022). The articles presented more than 3 years over the previous 6 years were emphasized by coloring their flows.

Journal Citation Reports (JCRs) for 2021 were used to obtain the journal impact factors. No informed permission or ethical approval was needed for this research because the data and information were all secondary data that were accessed from the open database (WOSCC).

## Results

### Co-authorship: Countries, institutions, and authors

**Figure 1:**
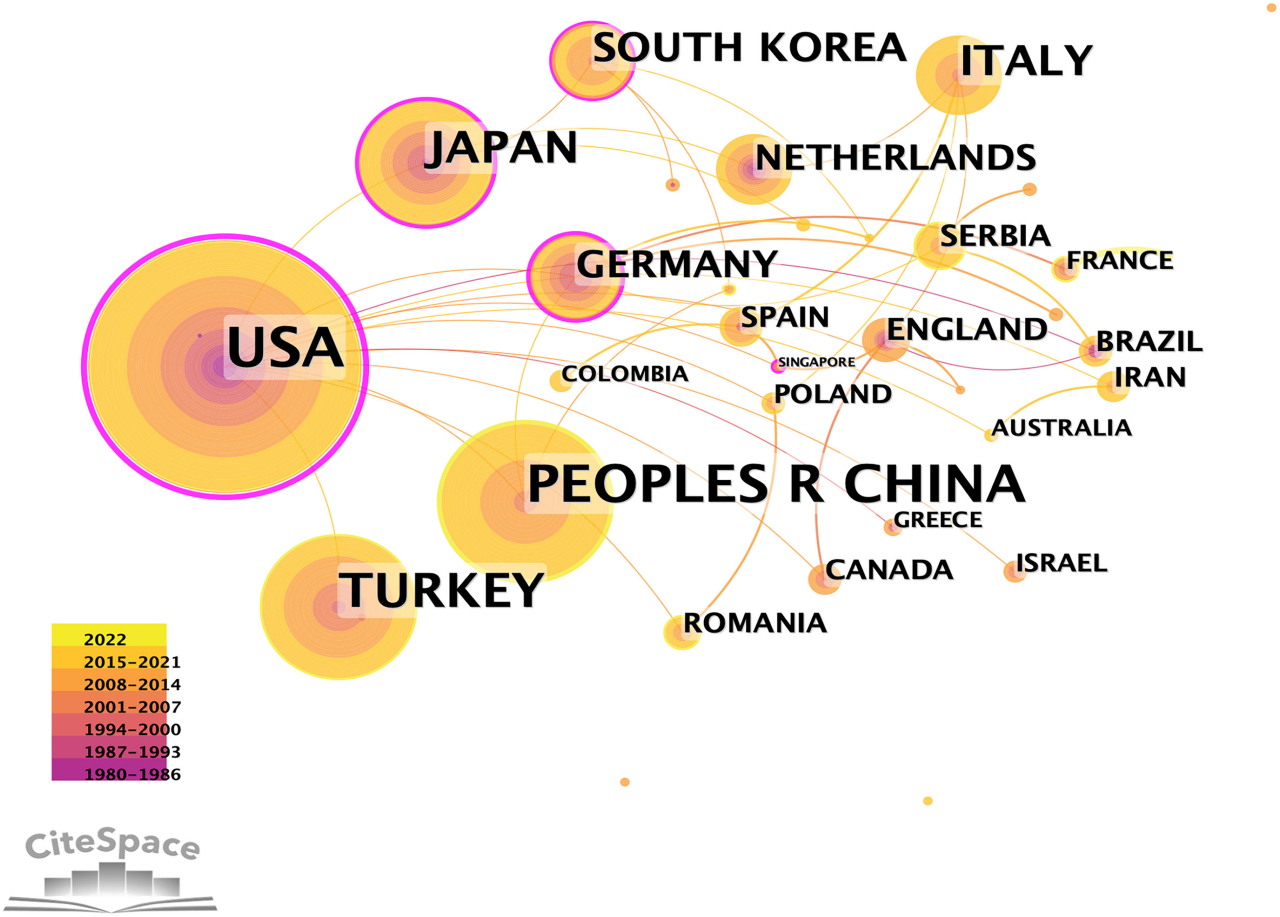
Co-authorship between countries with more than five publications.

**Figure 2:**
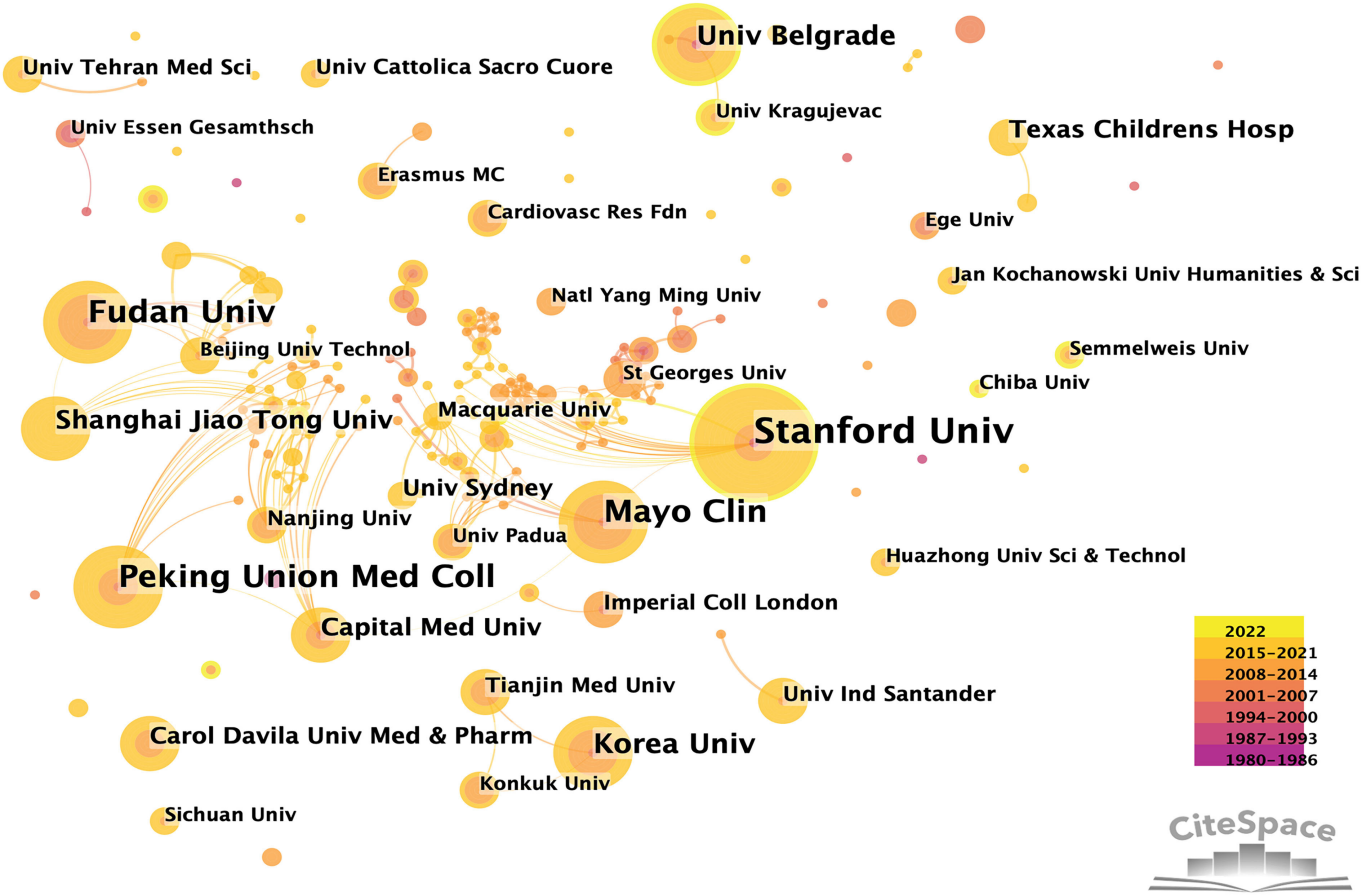
Co-authorship between institutions with more than four publications.

**Figure 3:**
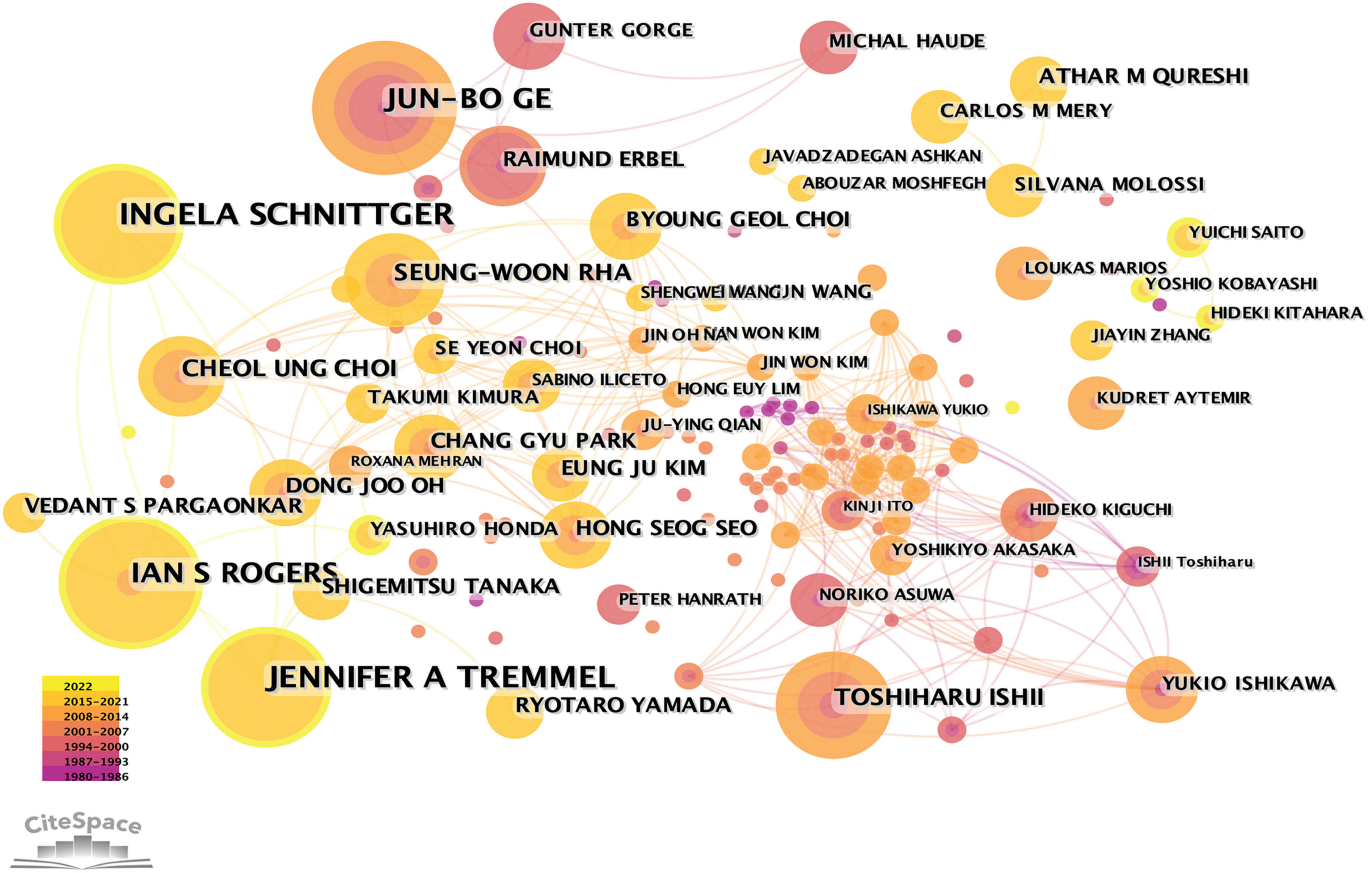
Co-authorship among authors with more than three publications.

In MB research, only 37 nations made significant contributions (Tables 1, 2 and Figure 1). The top five most-producing nations were the United States (150 articles), China (105 articles), Turkey (70 articles), Japan (48 articles), and Italy (41 articles). In terms of centrality, the top five countries were the United States (0.77), Japan (0.32), Germany (0.23), Italy (0.10), and the Netherlands (0.09). The co-authorship between institutions/authors is shown in Figures 2, 3. Stanford University published the most works, as indicated in Table 1, with 19 publications, followed by Fudan University (14 articles), Mayo Clinic (14 articles), Korea University (10 articles), University of Belgrade, Toho University, and China Academy of Chinese Medical Sciences (nine articles, respectively). Jennifer A. Tremmel, Ingela Schnittger, and Ian S. Rogers were the top three productive authors (Figure 3 and Table 1).

**Table 1:** Top 10 active countries, institutions, and authors.

**Rank**	**Categories**	**Records**
**Country**		
1	USA	150
2	People's Republic of China	105
3	Turkey	70
4	Japan	48
5	Italy	41
6	South Korea	31
7	Germany	26
8	The Netherlands	21
9	England	13
10	Serbia	11
**Institution**		
1	Stanford University (USA)	22
2	Fudan University (China)	14
3	Peking Union Medical College Hospital (China)	14
4	Mayo Clin (USA)	14
5	Korea University (South Korea)	10
6	University of Belgrade (Republic of Serbia)	9
7	Toho University (Japan)	9
8	Shanghai Jiao Tong University (China)	8
9	Capital Medical University (China)	7
10	Texas Children's Hosp (USA)	7
**Author**		
1	Jennifer A. Tremmel (USA)	17
2	Ingela Schnittger (USA)	16
3	Ian S. Rogers (USA)	15
4	Seung-Woon Rha (South Korea)	9
5	Toshiharu Ishii (Japan)	9
6	Vedant S. Pargaonkar (USA)	9
7	Jun-Bo Ge (China)	9
8	Cheol Ung Choi (South Korea)	8
9	Dong Joo Oh (South Korea)	7
10	Chang Gyu Park (South Korea)	7

Countries, institutions, and authors were ranked separately. The countries of institutions and authors were shown in brackets.

**Table 2:** Top 10 countries with high centrality value.

**Rank**	**Country**	**Centrality**
1	USA	0.77
2	Japan	0.32
3	Germany	0.23
4	Italy	0.10
5	The Netherlands	0.09
6	Russia	0.08
7	South Korea	0.07
8	Serbia	0.06
9	Spain	0.06
10	Brazil	0.06

### Co-occurrence analysis: Keywords

**Figure 4:**
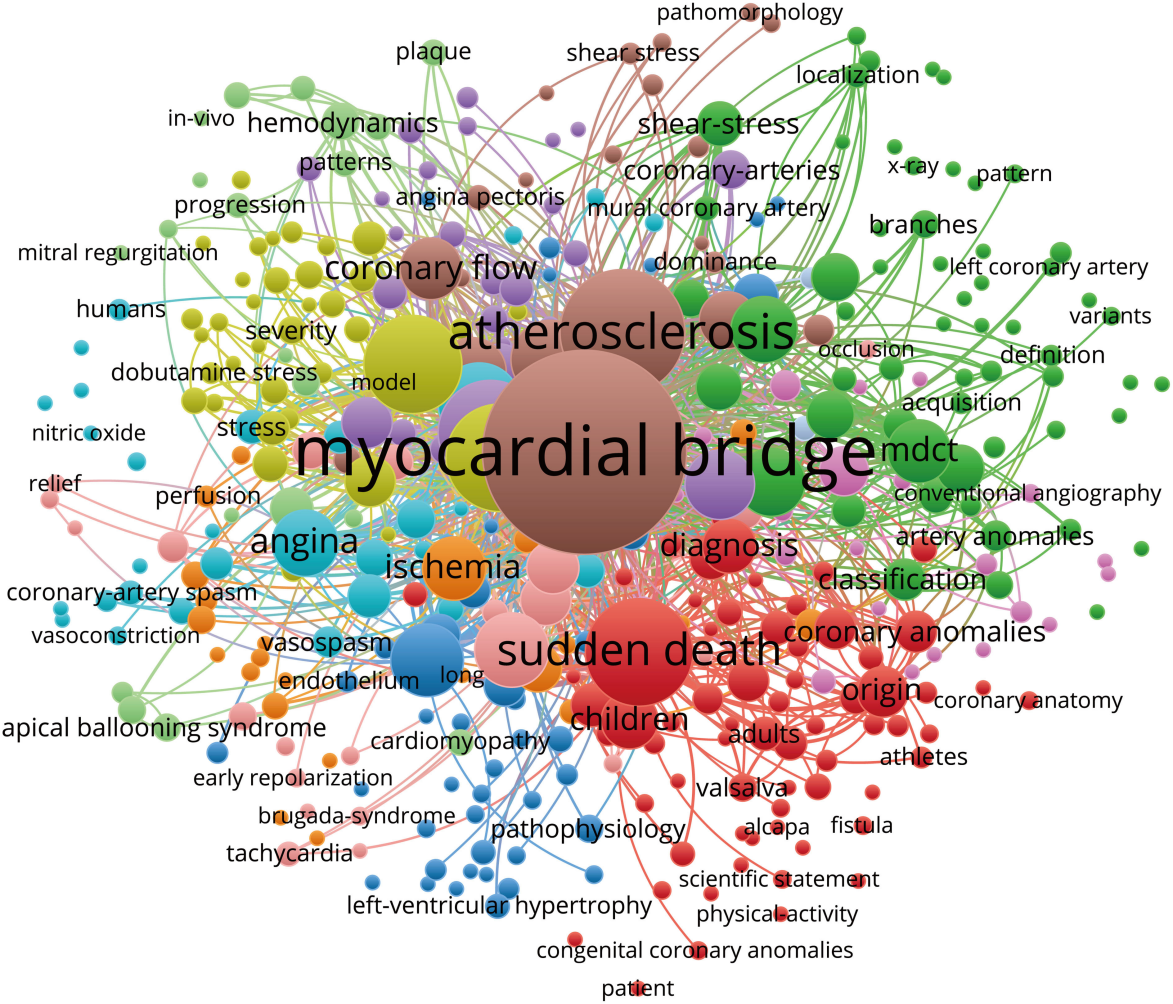
Keywords co-occurrence map of publications on the myocardial bridge.

**Figure 5:**
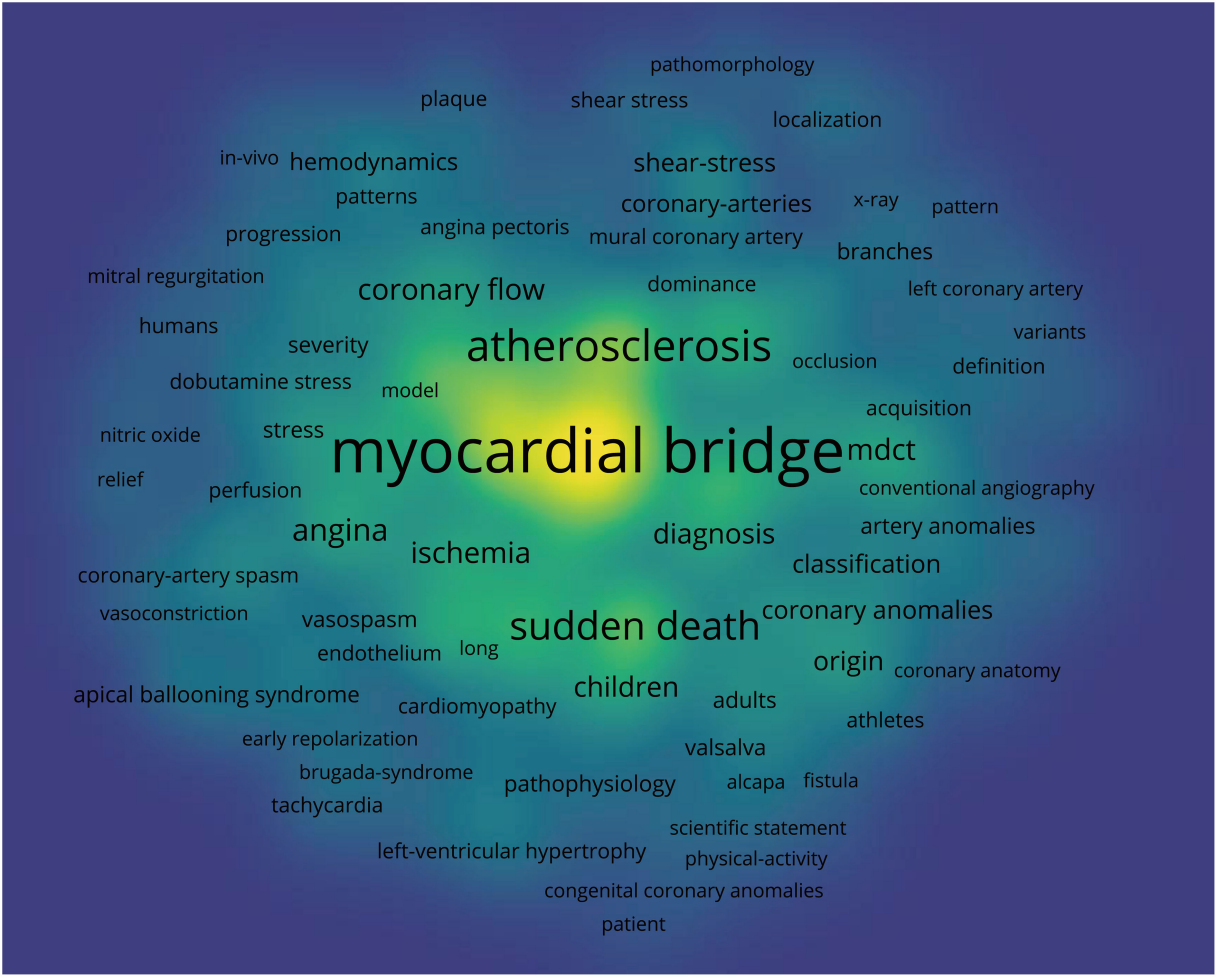
Keywords density visualization of the myocardial bridge.

Figure 4 shows the co-occurrence map of keywords on MB drawn by the VOS viewer. In addition, we created a keyword density visualization map (Figure 5). The following top 10 keywords appeared more than 50 times: myocardial bridge (372 records), mortality (140 records), coronary angiography (126 records), descending coronary artery (105 records), sudden death (105 records), infarction (99 records), intracoronary ultrasound (86 records), ultrasound (61 records), artery (57 records), and hypertrophic cardiomyopathy (50 records) (Table 3).

**Table 3:** Top 10 keywords in terms of records in myocardial bridge research.

**Rank**	**Keywords**	**Records**	**Rank**	**Keywords**	**Records**
1	Myocardial bridge	372	11	Disease	49
2	Atherosclerosis	140	12	Coronary artery disease	47
3	Coronary angiography	126	13	Exercise	47
4	Descending coronary artery	105	14	Coronary cta	43
5	Sudden death	105	15	Coronary artery	42
6	Infarction	99	16	Prevalence	42
7	Intracoronary ultrasound	86	17	Angina	40
8	Ultrasound	61	18	Coronary artery	39
9	Artery	57	19	Computed tomography	37
10	Hypertrophic cardiomyopathy	50	20	Ischemia	37

### Co-citation: References, journals, and authors

References cited simultaneously by two different publications were called co-cited references. The co-cited references yielded 10 co-cited authors and periodicals (Supplementary Figure). Tables 4, 5 include a list of the top 10 references, journals, and authors that were co-cited together. A total of 6,475 references were cited in 630 articles, according to the co-cited references map. There were 10 articles cited more than 100 times, up to 216 times. The co-cited journals map revealed that 630 publications had citations from 1,558 journals. Circulation (1,665 records) was in first place among the top 10 referenced journals (Table 4), followed by the Journal of the American College of Cardiology (1,119 records), American Heart Journal (807 records), European Heart Journal (704 records), and American Journal of Cardiology (694 records). References in the 630 articles were from a total of 4,937 authors. The top 10 authors in MB studies are shown in Table 4. The top one among them was Jun-Bo Ge, who had 332 records, followed by Paolo Angelini (274 records), Stefan Moehlenkamp (222 records), Yukio Ishikawa (177 records), and Toshiharu Ishii (162 records).

**Table 4:** Top 10 journals and authors with the highest citations.

**Rank**	**Journals**	**IF (2021)**	**JCR**	**Citations**	**Authors**	**Citations**
1	Circulation	39.918	Q1	1,665	Jun-Bo Ge	332
2	Journal of the American College of Cardiology	27.203	Q1	1,119	Paolo Angelini	274
3	American Heart Journal	5.099	Q2	807	Stefan Moehlenkamp	222
4	European Heart Journal	35.855	Q1	704	Yukio Ishikawa	177
5	American Journal of Cardiology	3.133	Q3	694	Toshiharu Ishii	162
6	International Journal of Cardiology	4.039	Q2	438	Ernst R. Schwarz	156
7	Catheterization and cardiovascular diagnosis^*^	N/A	N/A	382	Jacques Noble	153
8	Heart	7.365	Q1	305	Jorge R. Alegria	143
9	British Heart Journal^*^	N/A	N/A	263	Azorides R Morales	122
10	Chest	10.262	Q1	247	John R. Kramer	119

* These journals have shut down and have no IF and JCR in 2021.

**Table 5:** Top 10 references with highest citations.

**Rank**	**Reference**	**Authors**	**Year**	**Citations**
1	Update on Myocardial Bridging	Stefan Moehlenkamp	2002	216
2	Myocardial Bridging and Milking Effect of the Left Anterior Descending Coronary Artery: Normal Variant or Obstruction?	Jacques Noble	1976	144
3	Myocardial bridging	Jorge R. Alegria	2005	143
4	Comparison of Intravascular Ultrasound and Angiography in the Assessment of Myocardial Bridging	Jun-Bo Ge	1994	136
5	Myocardial Bridges: A Review	Paolo Angelini	1983	132
6	New signs characteristic of myocardial bridging were demonstrated by intracoronary ultrasound and Doppler	Jun-Bo Ge	1999	125
7	Clinical significance of isolated coronary bridges: Benign and frequent condition in the left anterior descending artery	John R. Kramer	1982	111
8	Symptomatic Myocardial Bridges: Overview of Ischemic Mechanisms and Current Diagnostic and Treatment Strategies	Martial G. Bourassa	2003	110
9	Myocardial bridges: morphological and functional aspects	Alberto G. Ferreira Jr	1991	102
10	The Mural Coronary	Eva R. Geiringer	1951	101

**Figure 6:**
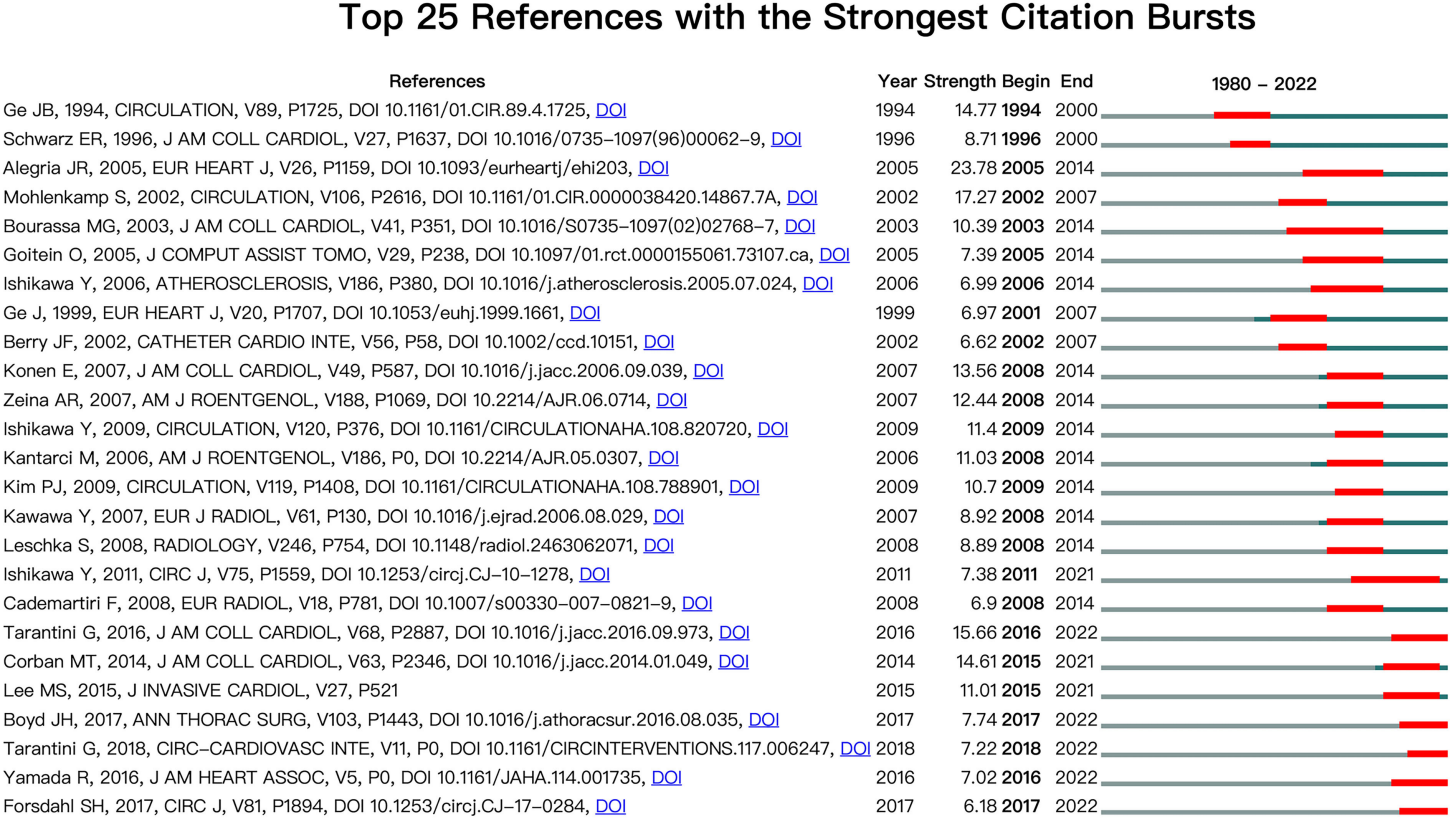
CiteSpace visualization map of the top 25 references with the strongest citation bursts involved in the myocardial bridge. Two articles, authored by Tarantini, began to burst after 2015.

Finding the references with the strongest citation bursts might help researchers identify hot subjects that are suddenly becoming more popular in a certain field and changes in the direction of their study. The references with the strongest citation bursts were examined. We especially concentrated on the references that started to burst after 2015 among the top 25 references with the strongest citation bursts (Figure 6). Two articles by Tarantini were obtained with a burst strength of 15.66 and 7.22. Tarantini et al. proposed that, when compared with fractional flow reserve (FFR), physiological assessment of MBs with instantaneous wave-free ratio (iFR) appears to be more consistent with patients' symptoms and the results of noninvasive tests (9).

**Figure 7:**
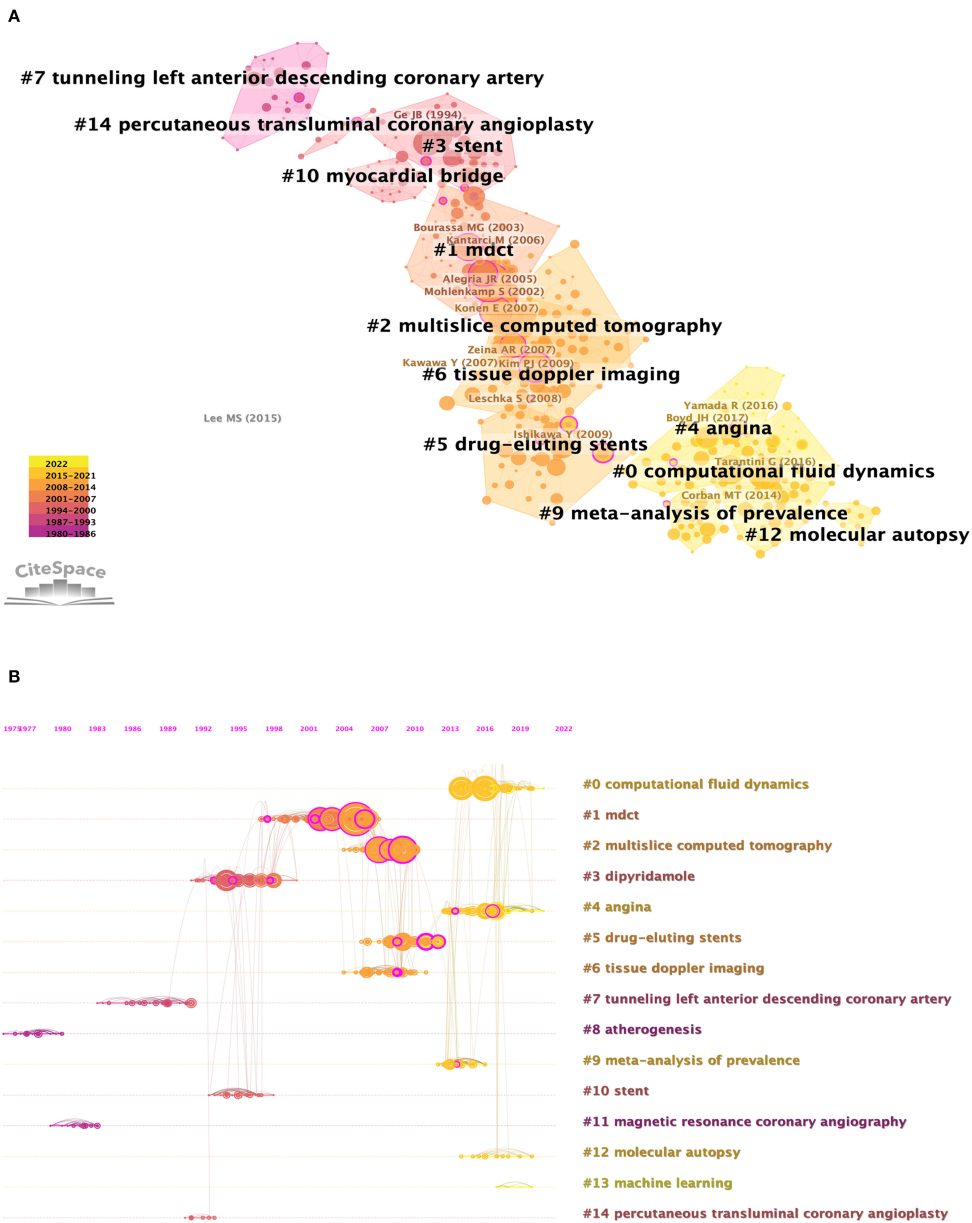
Analysis of co-citation references in the field of the myocardial bridge. **(A)** The network map of co-citation clusters. Fourteen clusters with different research topics were formed, reflecting in different colors on the map. **(B)** The timeline view of co-citation clusters. Each horizontal row represented a cluster, and each node presented by a “tree ring” on the line represented a study. The line between the nodes reflected the co-citation relationship between the two studies, and the size of the node meant the number of co-cited times. Cluster #0 computational fluid dynamics, #4 angina, #9 meta-analysis of prevalence, #12 molecular autopsy, and #13 machine learning were the most recent research directions.

**Figure 8:**
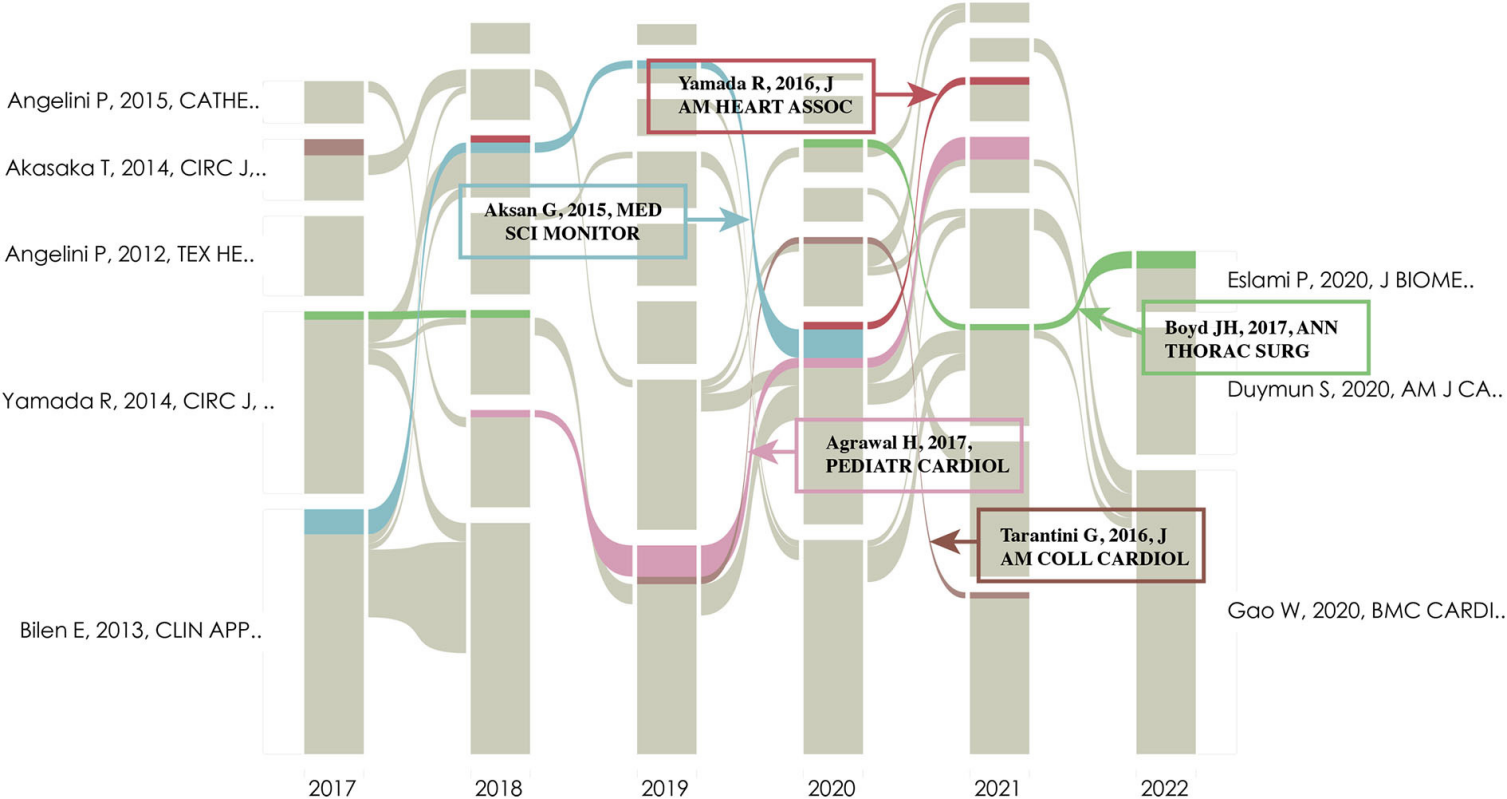
Alluvial flow map of co-cited references in the last 6 years. Each line represented a study, and colored and continuous lines referred to articles that had been cited more than 3 years in the past 6 years.

In the last 40 years, there have been 14 major study subjects that have been concentrated in the area of MB, as illustrated in Figure 7A, where 14 clusters of varying colors and sizes were developed. The chronology modifications of these clusters are also shown in Figure 8B, which reveals that the most recent areas are clusters #0, #4, #9, #12, and #13. In Table 6, we provided details for each cluster. The 14 clusters' silhouettes, which varied from 0.841 to 1.000, showed that their homogeneity was considerably greater. In addition, for clusters that have just emerged, clusters #12 and #13 had relatively few articles, which demonstrated that the studies in these domains were still immature. Furthermore, clusters #8 (“sudden death”) and #11 (“atherogenesis”) had the earliest average publication year among their members (1978 and 1981, respectively), indicating that they were early research topics in this area. In Supplementary Tables 2, 3, the top five referred and referring references are displayed in clusters #0, #4, #9, #12, and #13. Tarantini, Boyd, Migliore, Yuan, Ibrahim, and Deseive's works garnered the most citations in each of the aforementioned clusters. The alluvial diagram in Figure 8 shows the most commonly referred articles over the preceding 6 years, and five of them (Aksan, 2015, MED SCI MONITOR; Yamada, 2016, J AM HEART ASSOC; Agrawal, 2017, PEDIATR CARDIOL; Boyd, 2017, ANN THORAC SURG; and Tarantini, 2016, J AM COLL CARDIOL) were cited more than 5 years from 2017 to 2022, with two of them were related to computational fluid dynamics (Tarantini and Agrawal), two were associated with angina (Yamada and Boyd), and one was associated with meta-analysis of prevalence. The top-cited article in each cluster showed the rising trend of a certain study direction. Tarantini, Boyd, Migliore, and Yuan SM's publications were the most referenced articles in clusters #0, #4, #9, and #12, suggesting their significant contribution in the specific study direction (Supplementary Tables 2, 3).

**Table 6:** Details of clusters.

**Cluster ID**	**Size**	**Silhouette**	**Mean (Year)**	**Label (LLR)**
0	63	0.865	2016	Computational fluid dynamics
1	63	0.914	2002	MDCT (multi-detector tomography)
2	54	0.947	2007	Multi-slice computed tomography
3	44	0.972	1995	Dipyridamole
4	37	0.958	2016	Angina
5	35	0.840	2009	Drug-eluting stents
6	35	0.953	2008	Tissue doppler imaging
7	28	0.998	1987	Tunneling left anterior descending coronary artery
8	24	1.000	1978	Sudden death
9	23	0.986	2013	Meta-analysis of prevalence
10	21	0.944	1995	Stent
11	19	1.000	1981	Atherogenesis
12	9	0.990	2017	Molecular autopsy
13	6	1.000	2017	Machine learning
14	5	0.980	1992	Percutaneous transluminal coronary angioplasty

## Discussion

**Figure 9:**
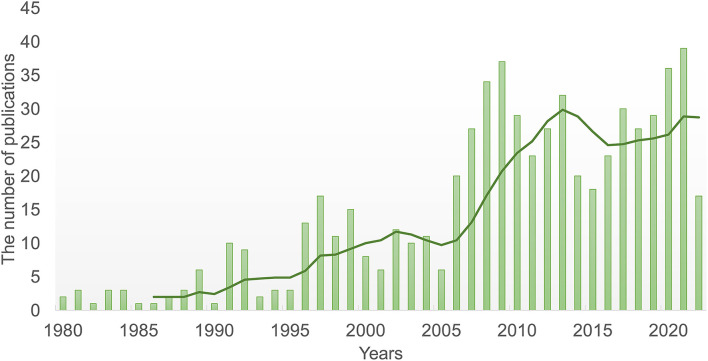
Trends of the myocardial bridge published over the past 43 years.

There are only roughly 630 articles discussing MB in the previous 43 years, owing to the low rate and wide range of clinical manifestations. An erratic growth tendency in articles indicates rising interest in MB (Figure 9).

The top 10 active countries consist of six European countries, three Asian countries, and the United States, accounting for 81.9% of the total literature (Table 1). Among them, the United States has more than two times the number of publications as Turkey, which ranks third. Stanford University, which is located in the United States, has the highest rating of centrality on MB. As a result, the United States had a central role in MB around the world (Table 2). However, although China has published the second greatest number of literature, its centrality was low.

According to the top 10 most popular journals, 40% (4/10) of the journals had an impact factor of more than 10, ranked in the JCR Q1 zone (Table 4). These journals were Circulation (IF_2021_ = 39.918), Journal of the American College of Cardiology (IF_2021_ = 27.203), European Heart Journal (IF_2021_ = 35.855), and Chest (IF_2021_ = 10.262).

The second most frequent co-cited reference was published by Noble et al. in 1976. They found that during tachycardia, patients with a systolic grade 3 milking effect in the left anterior descending (LAD) coronary artery may result in angina and anterior wall ischemia due to the severe obstruction of LAD by analyzing the hemodynamic data during a 60-watt supine ergocycle exercise test (10).

The reviews published in 2002 by Stefan Moehlenkamp et al. and in 2015 by Alegria et al. were the most and third most often mentioned references (Table 5). The articles highlighted clinically important features of myocardial bridging, with a focus on morphological and hemodynamic changes and how they are represented in imaging modalities. In angiography, the “milking effect” or “step down-step up” phenomenon provides little information on the myocardial functional effects. The morphological and functional aspects of MB can be observed and measured using intravascular ultrasound (IVUS), intracoronary Doppler ultrasonography (ICD), and intracoronary pressure devices (4, 11).

In Table 3, we can see that atherosclerosis, coronary angiography, descending coronary artery, and sudden death are closely related to MB. MBs are most commonly (70–98%) localized in the left anterior descending coronary artery (12). In addition to its association with myocardial ischemia, acute myocardial infarction, hypertrophic cardiomyopathy, cardiac arrhythmia, atrioventricular block, and thrombosis, its relations to atherosclerosis and sudden death have aroused much study interest. Earlier research suggested that MB may trigger or accelerate the evolution of atherosclerosis near the tunneled segment's entry (4, 13–15). However, more recent studies have failed to demonstrate a link between MB and proximal atherosclerosis (16–18). Previous research suggested that low shear stress near the bridge may contribute to the formation of atherosclerotic plaques, whereas high shear stress within the tunneled segment may play a protective role, which may explain the mechanism of atherosclerosis in the proximal segment to the bridging site (19). A recent case–control study using coronary CT angiography (CCTA) found no significant difference in atherosclerotic plaque volumes and compositions in the proximal LAD with or without MB (17). Two recent cohorts even showed that MB might operate as a possible preventive factor against severe obstructive atherosclerosis across the coronary artery system (16, 18). It is yet unknown how the trans-bridging segment wall shear stress gradient influences the formation of proximal atherosclerotic lesions. Articles by Dou et al. was the only one to cite every member of cluster #13 titled “Machine Learning,” and they used data distracted from coronary plaque in CCTA to predict major adverse cardiovascular events (MACEs) in patients with suspected CAD. Interestingly, they discovered MB to be a protective factor (20).

As technology advances, a variety of invasive and noninvasive procedures for assessing MB can be utilized. MB was originally noticed in 1951 when an in-depth examination of postmortem samples was reported, but clinical interest and systematic research were sparked in the 1970s by an observed link of MB with myocardial ischemia (10, 21, 22). The characteristic image of deep MB was discovered to be coronary angiography with the “milking effect” caused by systolic compression of the tunneled segment (10). However, in individuals with thin bridges, the milking effect may be missed, and numerous novel imaging approaches have been developed to detect a bridge on morphological, hemodynamic, and functional evaluation (23–30). The typical intravascular ultrasound (IVUS) finding is a “half-moon” sign, which represents an echolucent area present immediately adjacent to the vessel lumen that persists throughout the cardiac cycle and is demonstrated by Yamada et al. to represent a muscle band overlying the tunneled arterial segment (29). Optical coherence tomography (OCT) can also detect susceptible plaque and offer a more thorough view of the architecture of the coronary arteries (31). Using pressure wire methods like fractional flow reserve (FFR), a distinctive velocity pattern of the MB segment may be identified, which can be utilized to analyze MB functionally and physiologically. Over the last 5 years, the instantaneous wave-free ratio (iFR) has become more widely employed in the functional assessment of MB. Tarantini et al. demonstrated that iFR is superior to FFR (9).

Unlike IVUS, OCT, and FFR, CCTA is a noninvasive test that also increases the detection rate of MB by up to 58% (24). It was widely used to visualize the coronary artery lumen and surrounding structures in three dimensions (24). CT-derived FFR has been used to examine MB; however, it may suffer from some of the same drawbacks as CCTA and conventional invasive FFR (32). As shown in Figure 7B, dipyridamole, as the name of cluster #3, has also been studied. Dipyridamole ^20^1Tl Myocardial SPECT can be used to assess the myocardial ischemia of a patient with MB, and it has played an important role in clinical decision-making (33).

Another area that requires special attention is molecular autopsy, which was recognized as one of the most recent regions in MB by the designation of cluster #12 (Figure 7B). Currently, postmortem genetic testing in the cases of suddenly died young persons may frequently contribute significantly to determining the cause of death (34). MB is a frequent congenital defect observed in up to 85% of forensic autopsies (35). The prevalence of MB has been observed to be 21–41% in patients with hypertrophic cardiomyopathy (36). The features of the MB discovered by Simone Grassi et al. as well as the *in silico* predictions about the SLMAP gene variation, imply that these results might have produced a fatal arrhythmia, which requires further investigation (37).

The name of cluster #9 is a meta-analysis of prevalence. The prevalence of MB in different studies ranged from 0.004 to 80% (38, 39). Three of the top five articles citing references in cluster #9 were talking about the prevalence of MB. Hostiuc et al. included 120 studies to analyze and discovered that the estimated prevalence was 19% (17–21%), with LAD having the greatest overall frequency of 82% (40).

The article covering most of the articles in cluster #4, named angina, was published by Okada et al. They investigated the effect of MB on life-threatening ventricular arrhythmia (LTVA) after a median of 4.5 (2.2–7.1) years of follow-up in patients with implanted cardioverter defibrillator (ICD). They revealed that patients with MB had significantly higher rates of LTVA and a higher prevalence of vasospastic angina than patients without MB. It may account for some potential mechanisms for bad prognosis in patients with myocardial infarction/ischemia with non-obstructive coronary arteries (MINOCAs) (41). MB may be the major etiology of angina in MINOCA, given the high proportion (58%) of patients with MB detected by IVUS in patients suffering from angina but without the absence of obstructive CAD (42). Although most MBs were considered a benign cardiovascular anomaly, the potential poor prognosis of some symptomatic patients should be paid more attention, and the evaluation of MB seems to improve the identification of high-risk individuals in case of the occurrence of LTVA or sudden death (41).

There are still no guideline recommendations for MB due to the lack of randomized clinical trials. Although its medical management and surgical treatment did not change much during these four decades, the studies evaluating their prognosis never stopped. In general, medical therapy should be regarded as the initial therapeutic strategy, with the beta-blockers and/or non-dihydropyridine calcium channel blockers serving as first-line treatment and ivabradine as the second-line choice for those who do not tolerate beta-blockers or calcium channel blockers (43–46). As shown in Figure 7, PCI with a stent has gained much attention for a long time due to its availability and ease and the historical effectiveness for patients who are suffering from refractory symptoms after receiving appropriate anti-anginal medication. However, it has previously demonstrated higher rates of in-stent restenosis for bare-metal stents than drug-eluting stents (DESs) at 1 year (75 vs. 25%) (47). Given the high rates of in-stent restenosis and some other complications like coronary perforation and stent fracture, PCI should be the last option for patients with MB who are not surgical candidates, having a predilection for high radial force second-generation DES (48, 49).

Binet et al. were the first to describe surgical unroofing (or myotomy) for persons who were unresponsive to treatment in 1975 (50). According to a prospective cohort of 50 adults with a mean 6.6-month follow-up, Boyd work, which was published in Annals of Thoracic Surgery in 2017 and is also the most prominent study in cluster #4, showed that surgical unroofing may be performed for patients with LAD-MB as an independent treatment with considerable improvement and no serious problems or fatalities in symptoms afterward (51). In addition to the potential short-term complications like ventricular wall perforation, artery perforation, and ventricular aneurysm formation et al. (52) a significant frequency of late recurring chest discomfort (up to 60%) following successful unroofing in adult patients within 3 years was reported by Hemmati et al. (53). Coronary artery bypass grafting (CABG) was reported as another important surgical option for patients with MB (54, 55). However, due to the high risk of the left internal mammary artery (LIMA) graft failure in thin or short MB, CABG was preferable for patients with deep and/or extensive MB and/or with atherosclerosis occurring at the proximal tunnel segment (56). It may be explained by the competitive flow in the native coronary artery after CABG. Given the graft occlusion rate of 60% in the LIMA group vs. 15.8% in the SVG group, bypassing with a saphenous vein graft (SVG) may be a better option than with LIMA (55). To resolve the problem of competitive flow, Zhang et al. developed a novel surgical procedure named MB bypass grafting (MBBG) for extensive MB by using a free LIMA to bridge from the proximal to the distal end of the tunnel artery (57). More clinical trials and follow-ups are needed to establish the efficacy.

This research has certain limitations. To begin, we collected scientific articles from WoSCC but excluded other databases such as Google Scholar and PubMed, and the language was limited to English, which may have resulted in bias. Second, because the material we downloaded initially was not the whole text, some relevant facts or perspectives may have been excluded. Nonetheless, our research is based on all objectively gathered data, with no supervisor bias. Third, we have tried our best to replace the authors' full names in analyzing co-authorship, but in analyzing co-reference, the bibliometrics software was unable to identify the authors with the same name owing to the similar abbreviations of certain authors' names in references. Loss of accuracy may still be inevitable in co-reference analysis. Finally, there may still be some literature not being read and analyzed by authors, losing some more meaningful research directions.

## Conclusion

In our study, we found that MB research has shown a variable growth tendency over the previous four decades. Our goal was to review previous studies in the field of MB, understand the context of MB research, and recommend new directions for future study. Standard guidelines for the optimum diagnosis and therapy of MB require more collaboration and exchange between countries and organizations. The current focus of MB research in cardiovascular science is on the prevalence and prognosis, mechanism of atherosclerosis, hemodynamics, and molecular autopsy, all of which will be the focus of future studies.

## Data availability statement

Publicly available datasets were analyzed in this study. This data can be found here: webofscience.com.

## Author contributions

LC and W-YY: conceptualization, formal analysis, and software. B-LW and M-XG: data curation. YY: investigation, project administration, and resources. LC, W-YY, RL, and X-HD: methodology. LC: writing—original draft. YY, M-XG, and B-LW: writing—review and editing. All authors contributed to the article and approved the submitted version.
