# Supplementary material for: A bibliometric analysis on the progress of myocardial bridge from 1980 to 2022
Source: Front Cardiovasc Med. 2023 Jan 6;9:1051383. doi: 10.3389/fcvm.2022.1051383 (PMC9853984; doi:10.3389/fcvm.2022.1051383)
Supplement: Supplementary file 1 [file Data_Sheet_1.PDF]

Supplementary TABLE 1. Number of publication associated with myocardial bridge from 1980 to July 31, 2022.

Years	The number of publications
1961	1
1963	1
1971	1
1976	1
1977	2
1978	1
1980	2
1981	3
1982	1
1983	3
1984	3
1985	1
1986	1
1987	2
1988	3
1989	6
1990	1
1991	10
1992	9
1993	2
1994	3
1995	3
1996	13
1997	17
1998	11

Years	The number of publications
1999	15
2000	8
2001	6
2002	12
2003	10
2004	11
2005	6
2006	20
2007	27
2008	34
2009	37
2010	29
2011	23
2012	27
2013	32
2014	20
2015	18
2016	23
2017	30
2018	27
2019	29
2020	36
2021	39
2022	17

Supplementary TABLE 2. The top 5 papers in cluster #0, 4, 9, 12 and 13.

Cluster	Citation	Author	Publication year	Title	Journal	DOI
**0**	33	Tarantini G	2016	Left Anterior Descending Artery Myocardial Bridging	Journal of the American College of Cardiology	10.1016/j.jacc.2016.09.973
**0**	28	Corban MT	2014	Myocardial bridging is associated with coronary atherosclerosis in the segment proximal to the site of bridging	Journal of the American College of Cardiology	10.1016/j.jacc.2014.01.049
**0**	14	Tarantini G	2018	Unmasking Myocardial Bridge-Related Ischemia by Intracoronary Functional Evaluation	Circulation-Cardiovascular Interventions	10.1161/CIRCINTERVENTIONS.117.006247
**0**	8	Uusitalo V	2015	the Functional Effects of Intramural Course of Coronary Arteries and its Relation to Coronary Atherosclerosis	JACC-Cardiovascular Imaging	10.1016/j.jcmg.2015.04.001
**0**	7	Saito Y	2017	Relation between severity of myocardial bridge and vasospasm	International Journal of Cardiology	10.1016/j.ijcard.2017.07.002
**4**	18	Boyd JH	2017	Surgical Unroofing of Hemodynamically Significant Left Anterior Descending Myocardial Bridges	Annals of Thoracic Surgery	10.1016/j.athoracsur.2016.08.035
**4**	15	Yamada R	2016	Functional Versus Anatomic Assessment of Myocardial Bridging by Intravascular Ultrasound: Impact of Arterial Compression on Proximal Atherosclerotic Plaque	Journal of the American Heart Association	10.1161/JAHA.114.001735
**4**	12	Forsdahl SH	2017	Myocardial Bridges on Coronary Computed Tomography Angiography	Circulation Journal	10.1253/circj.CJ-17-0284
**4**	10	Lee BK	2015	Invasive Evaluation of Patients with Angina in the Absence of Obstructive Coronary Artery Disease	Circulation	10.1161/CIRCULATIONAHA.114.012636
**4**	9	Lin S	2013	A Novel Stress Echocardiography Pattern for Myocardial Bridge with Invasive Structural and Hemodynamic Correlation	Journal of the American Heart Association	10.1161/JAHA.113.000097
**9**	9	Migliore F	2013	LAD Coronary Artery Myocardial Bridging and Apical Ballooning Syndrome	JACC-Cardiovascular Imaging	10.1016/j.jcmg.2012.08.013
**9**	7	Nakaura T	2014	Myocardial bridging is associated with coronary atherosclerosis in the	Journal of Cardiology	10.1016/j.jjcc.2013.07.005

				segment proximal to the site of bridging		
**9**	6	Niu YJ	2013	Clinical value of the correlations of mural coronary artery compression extent with myocardial bridge length and thickness evaluated by 128-slice CT	Experimental and therapeutic Medicine	10.3892/etm.2012.879
**9**	6	Stiermaier T	2014	Frequency and Significance of Myocardial Bridging and Recurrent Segment of the Left Anterior Descending Coronary Artery in Patients With Takotsubo Cardiomyopathy	American Journal of Cardiology	10.1016/j.amjcard.2014.07.040
**9**	6	Ma ES	2013	Assessment of Myocardial Bridge and Mural Coronary Artery Using ECG-Gated 256-Slice CT Angiography: A Retrospective Study	Scientific World Journal	10.1155/2013/947876
**12**	7	Yuan SM	2016	Myocardial Bridging	Brazilian Journal of Cardiovascular Surgery	10.5935/1678-9741.20150082
**12**	3	Priori SG	2015	2015 ESC Guidelines for the management of patients with ventricular arrhythmias and the prevention of sudden cardiac death	European Heart Journal	10.1093/eurheartj/ehv316
**12**	3	Kiess A	2018	Symptomatic myocardial bridging: a frequently occurring coronary variation can cause severe myocardial ischaemia in affected children with underlying cardiac conditions	Cardiology in the Young	10.1017/S1047951118000409
**12**	2	Elliott PM	2014	2014 ESC Guidelines on diagnosis and management of hypertrophic cardiomyopathy	European Heart Journal	10.1093/eurheartj/ehu284
**12**	2	Basso C	2017	Guidelines for autopsy investigation of sudden cardiac death: 2017 update from the Association for European Cardiovascular Pathology	Virchows Archiv	10.1007/s00428-017-2221-0
**13**	1	Deseive S	2017	Improved 5-year prediction of all-cause mortality by coronary CT angiography applying the CONFIRM score	European Heart Journal-Cardiovascular Imaging	10.1093/ehjci/jew195
**13**	1	Bzdok D	2018	Statistics versus machine learning	Nature Methods	10.1038/nmeth.4642

**13**	1	andreini D	2017	Long-term prognostic impact of CT-Leaman score in patients with non-obstructive CAD: Results from the COronary CT Angiography EvaluatioN For Clinical Outcomes InteRnational Multicenter (CONFIRM) study	International Journal of Cardiology	10.1016/j.ijcard.2016.12.137
**13**	1	Abdelrahman KM	2020	Coronary Computed Tomography Angiography From Clinical Uses to Emerging Technologies: JACC State-of-the-Art Review	Journal of the American College of Cardiology	10.1016/j.jacc.2020.06.076
**13**	1	Danad I	2017	Diagnostic performance of cardiac imaging methods to diagnose ischaemia-causing coronary artery disease when directly compared with fractional flow reserve as a reference standard: a meta-analysis	European Heart Journal	10.1093/eurheartj/ehw095

Supplementary TABLE 3. The top 5 papers that cited the members of cluster #0, 4, 9, 12 and 13.

Cluster	Coverage	Author	Year	Citing paper title	Journal	DOI
**0**	11	Zhou F	2019	Fractional flow reserve derived from CCTA may have a prognostic role in myocardial bridging	European Radiology	10.1007/s00330-018-5811-6
**0**	10	Zhang M	2019	Longitudinal strain measured by two-dimensional speckle tracking echocardiography to evaluate left ventricular function in patients with myocardial bridging of the left anterior descending coronary artery	Echocardiography-A Journal of Cardiovascular Ultrasound and Allied Techniques	10.1111/echo.14357
**0**	10	Okada K	2020	Impact of Myocardial Bridge on Life-Threatening Ventricular Arrhythmia in Patients With Implantable Cardioverter Defibrillator	Journal of the American Heart Association	10.1161/JAHA.120.017455
**0**	9	Zhou F	2019	Diagnostic Performance of Machine Learning Based CT-FFR in Detecting Ischemia in Myocardial Bridging and Concomitant Proximal Atherosclerotic Disease	Canadian Journal of Cardiology	10.1016/j.cjca.2019.08.026
**0**	8	Montone R	2021	Interplay Between Myocardial Bridging and Coronary Spasm in Patients With Myocardial Ischemia and Non-Obstructive Coronary Arteries: Pathogenic and Prognostic Implications	Journal of the American Heart Association	10.1161/JAHA.120.020535
**4**	10	Okada K	2020	Impact of Myocardial Bridge on Life-Threatening Ventricular Arrhythmia in Patients With Implantable Cardioverter Defibrillator	Journal of the American Heart Association	10.1161/JAHA.120.017455
**4**	9	Saito K	2022	Impact of myocardial bridge on non-culprit vessel lumen changes in patients with acute coronary syndrome	Heart and Vessels	10.1007/s00380-022-02130-y
**4**	9	Boyd J	2017	Surgical Unroofing of Hemodynamically Significant Left Anterior Descending Myocardial Bridges	Annals of Thoracic Surgery	10.1016/j.athoracsur.2016.08.035
**4**	7	Ermis P	2017	Stress echocardiography: An overview for use in pediatric and congenital cardiology	Congenital Heart Disease	10.1111/chd.12495

**4**	7	Saito K	2022	Influence of myocardial bridge on atherosclerotic plaque distribution and characteristics evaluated by near-infrared spectroscopy intravascular ultrasound	Heart and Vessels	10.1007/s00380-022-02083-2
**9**	16	Hostiuc S	2018	Myocardial Bridging: A Meta-Analysis of Prevalence	Journal of Forensic Sciences	10.1111/1556-4029.13665
**9**	11	Hostiuc S	2017	Cardiovascular consequences of myocardial bridging: A meta-analysis and meta-regression	Scientific Reports	10.1038/s41598-017-13958-0
**9**	9	Boyd J	2017	Surgical Unroofing of Hemodynamically Significant Left Anterior Descending Myocardial Bridges	Annals of Thoracic Surgery	10.1016/j.athoracsur.2016.08.035
**9**	5	Paul A	2016	Anomalous origins and branching patterns in coronary arteries - An angiographic prevalence study	Journal of the Anatomical Society of India	10.1016/j.jasi.2016.09.001
**9**	4	Arcari L	2017	Tortuosity, Recurrent Segments, and Bridging of the Epicardial Coronary Arteries in Patients With the Takotsubo Syndrome	American Journal of Cardiology	10.1016/j.amjcard.2016.09.055
**12**	7	Grassi S	2020	Genetic variants of uncertain significance: How to match scientific rigour and standard of proof in sudden cardiac death?	Legal Medicine	10.1016/j.legalmed.2020.101712
**12**	5	GRASSI S	2021	Sudden Death without a Clear Cause after Comprehensive Investigation: An Example of Forensic Approach to Atypical/Uncertain Findings	Diagnostics	10.3390/diagnostics11050886
**12**	2	KURATH-KOLLER S	2020	Wearable cardioverter-defibrillator as bridging to ICD in pediatric hypertrophic cardiomyopathy with myocardial bridging - a case report	Bmc Pediatrics	10.1186/s12887-020-02113-w
**12**	2	PELLICCI A A	2017	Are Olympic athletes free from cardiovascular diseases? Systematic investigation in 2352 participants from Athens 2004 to Sochi 2014	British Journal of Sports Medicine	10.1136/bjsports-2016-096961
**13**	6	DOU G	2022	Integrating Coronary Plaque Information from CCTA by ML Predicts MACE in Patients with Suspected CAD	Journal of Personalized Medicine	10.3390/jpm12040596

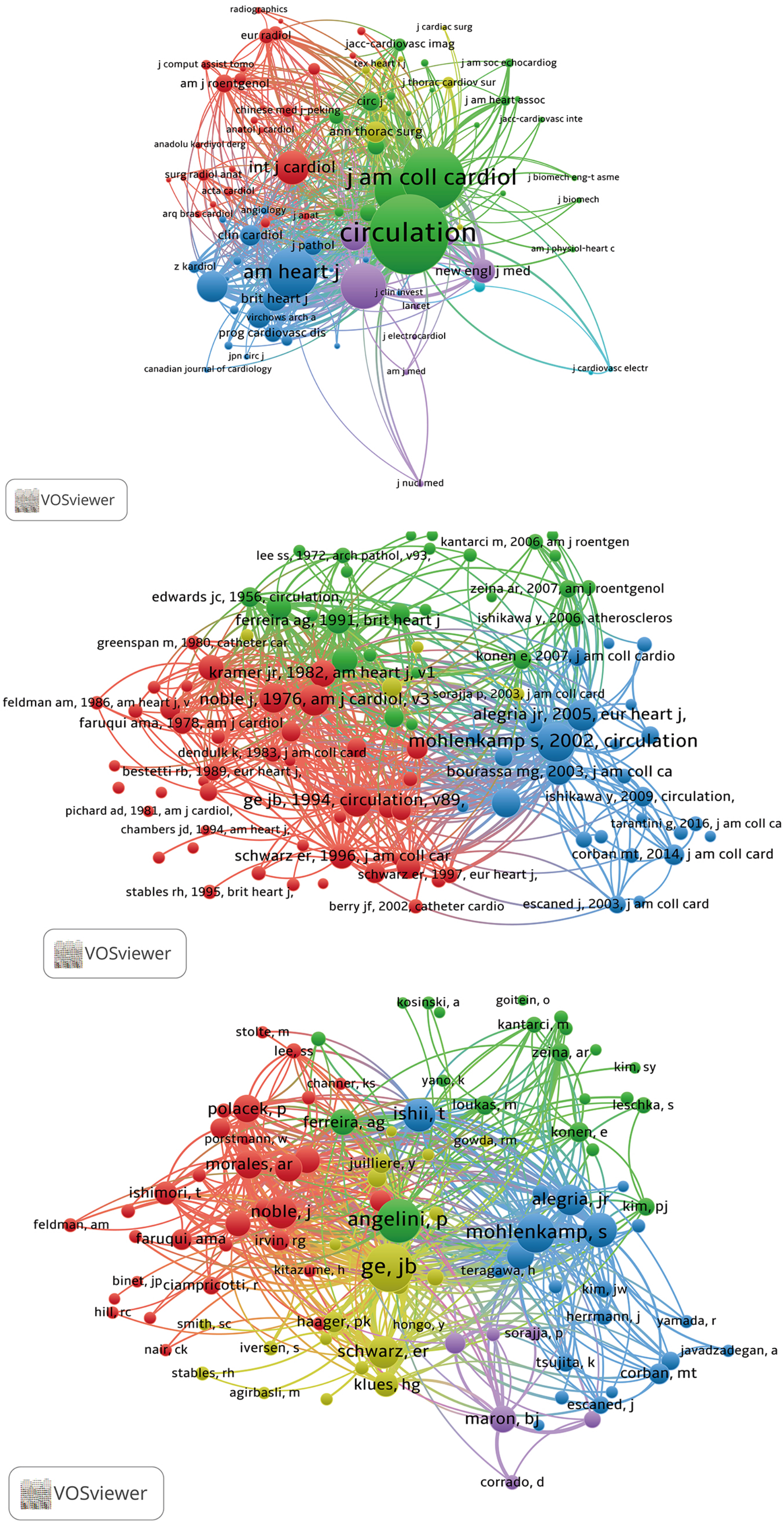

Supplementary figures.

Map of co-citations for top 100 most cited journals (A) /references (B)/ authors(C) .
